# Supplementary material for: Genomics of sex allocation in the parasitoid wasp Nasonia vitripennis
Source: BMC Genomics. 2020 Jul 20;21:499. doi: 10.1186/s12864-020-06904-4 (PMC7372847; doi:10.1186/s12864-020-06904-4)
Supplement: Supplementary file 5 — Additional file 5: Supplementary Figure 1. Hierarchical clustering of all 34 NVGRP lines based on the average pairwise FST values over all variable sites between lines. Plot shows clustering based on the complete linkage method in R package hclust. Supplementary Figure 2. Distribution of phenotypic values over all 26 tested NVGRP lines, for (a) sex ratio expressed as proportion males, and (b) clutch size. Supplementary Figure 3. Boxplot of sex ratio (proportion males) and clutch size among all 26 tested NVGRP lines. Plot shows the median, the box indicates the first and the third quartile, the whiskers indicate 1.5 times the interquartile range and values beyond that are labelled outliers (open circles). Supplementary Figure 4. Scatterplot of the mean clutch size and the mean sex ratio for the 25 tested NVGRP lines (excluding outlier line 62). Solid line shows the fit of the linear regression y = 0.224467–0.001477x. Supplementary Figure 5. Multipaneled plot showing the cumulative results of the SuperExactTest for association of the p-values of the sex ratio and clutch size GWAS per chromosome for windows of 25 kb, 50 kb, 100 kb, 200 kb and 400 kb. Black solid line shows observed overlap using GWAS p values for each SNPs, red solid line shows the most significant p-value for each window given from a distribution of 100 permutations. Significant overlap is observed for all window sizes, when the p-values of the association between the sex ratio and clutch size GWAS exceeds the p-values of the permutation. [file 12864_2020_6904_MOESM5_ESM.docx]

**Supplementary Material**

**Genomics of sex allocation in the parasitoid wasp *Nasonia vitripennis***

Bart A. Pannebakker^1*^, Nicola Cook^2^, Joost van den Heuvel^1^, Louis van de Zande^3^ & David M. Shuker^2^

**Supplementary Figures**

*Supplementary Figure 1.* Hierarchical clustering of all 34 NVGRP lines based on the average pairwise *F_ST_* values over all variable sites between lines. Plot shows clustering based on the complete linkage method in R package hclust.

a

b

*Supplementary Figure 2.* Distribution of phenotypic values over all 26 tested NVGRP lines, for (a) sex ratio expressed as proportion males, and (b) clutch size.

*Supplementary Figure 3.* Boxplot of sex ratio (proportion males) and clutch size among all 26 tested NVGRP lines. Plot shows the median, the box indicates the first and the third quartile, the whiskers indicate 1.5 times the interquartile range and values beyond that are labelled outliers (open circles).

*Supplementary Figure 4.* Scatterplot of the mean clutch size and the mean sex ratio for the 25 tested NVGRP lines (excluding outlier line 62). Solid line shows the fit of the linear regression y= 0.224467 -0.001477x.

*Supplementary Figure 5.* Multipaneled plot showing the cumulative results of the SuperExactTest for association of the p-values of the sex ratio and clutch size GWAS per chromosome for windows of 25kb, 50kb, 100kb, 200kb and 400kb. Black solid line shows observed overlap using GWAS p values for each SNPs, red solid line shows the most significant p-value for each window given from a distribution of 100 permutations. Significant overlap is observed for all window sizes, when the p-values of the association between the sex ratio and clutch size GWAS exceeds the p-values of the permutation.
